# Deformable image registration of dark‐field chest radiographs for functional lung assessment

**DOI:** 10.1002/mp.18023

**Published:** 2025-08-08

**Authors:** Fabian Drexel, Vasiliki Sideri‐Lampretsa, Henriette Bast, Alexander W. Marka, Thomas Koehler, Florian T. Gassert, Daniela Pfeiffer, Daniel Rueckert, Franz Pfeiffer

**Affiliations:** ^1^ Chair of Biomedical Physics, Department of Physics, School of Natural Sciences Technical University of Munich Garching Germany; ^2^ Munich Institute of Biomedical Engineering Technical University of Munich Garching Germany; ^3^ Institute for Diagnostic and Interventional Radiology, School of Medicine and Health, TUM Klinikum Technical University of Munich (TUM) Munich Germany; ^4^ Chair for AI in Healthcare and Medicine Technical University of Munich (TUM) and TUM University Hospital Munich Germany; ^5^ Philips Innovative Technologies Hamburg Germany; ^6^ Institute for Advanced Study Technical University of Munich Garching Germany; ^7^ Munich Center for Machine Learning (MCML) Munich Germany; ^8^ Department of Computing Imperial College London London UK

**Keywords:** dark‐field radiography, functional imaging, image registration, lung imaging

## Abstract

**Background:**

Dark‐field radiography of the human chest has been demonstrated to have promising potential for the analysis of the lung microstructure and the diagnosis of respiratory diseases. However, most previous studies of dark‐field chest radiographs evaluated the lung signal only in the inspiratory breathing state.

**Purpose:**

Our work aims to add a new perspective to these previous assessments by locally comparing dark‐field lung information between different respiratory states to explore new ways of functional lung imaging based on dark‐field chest radiography.

**Methods:**

We use suitable deformable image registration methods for dark‐field chest radiographs to establish a mapping of lung areas in distinct breathing states. After registration, we utilize an inter‐frame ratio approach to examine the local dark‐field signal changes and evaluate the gradient of the craniocaudal axis projections and mean lung field values to draw a quantitative comparison to standard chest radiographs and assess the relationship with the respiratory capacity.

**Results:**

Considering full inspiration and expiration scans from a clinical chronic obstructive pulmonary disease study, the registration framework allows to establish an accurate spatial correspondence (Median Dice score 0.95/0.94, mean surface distance 3.71/3.52 mm, and target registration error 6.10 mm) between dark‐field chest radiographs in different respiratory states and thus to perform a local signal change analysis. Compared to the utilization of standard chest radiographs, the presented approach benefits from the absence of bone and soft‐tissue structures in the dark‐field images, which move differently during respiration than the lung tissue. Our quantitative evaluation of the inter‐frame ratios demonstrates evidence of craniocaudal gradient‐sensitivity advantages concerning the relative vital lung capacity of the study participants in the dark‐field images (Spearman correlation coefficients: $r_{s,right}=0.55$, p<0.01 and $r_{s,left}=0.48$, p<0.01 compared to the attenuation image‐based gradient correlations $r_{s,right}=0.20$, p=0.16 and $r_{s,left}=0.40$, p<0.01). Moreover, our alternative lung field analysis approach provides insights into the distinct behavior of the dark‐field signal changes with the breathing capacity, which are in good agreement with the expected lung volume changes in the respective lung regions. In quantitative terms, this is reflected in a weak Spearman correlation ($r_{s,\mathrm{upper}}=0.30$, p=0.01) of the mean dark‐field signal ratio within the upper lung region, but strong correlations within the middle ($r_{s,\mathrm{middle}}=0.71$, p<0.01) and lower ($r_{s,\mathrm{lower}}=0.67$, p<0.01) lung region.

**Conclusions:**

Our regional characterization of lung dark‐field signal changes between the breathing states via deformable image registration provides a proof‐of‐principle that dynamic radiography‐based lung function assessment approaches may benefit from considering registered dark‐field images in addition to standard plain chest radiographs. This opens up new options for low‐dose and rapid lung ventilation assessment via dark‐field chest radiography that has the potential to improve lung diagnostics considerably.

## 1 INTRODUCTION

Dark‐field radiography is an imaging technique that allows to visualize micro‐structural features of an investigated sample.^1^, ^2^ In this imaging approach, the dark‐field contrast is formed through the mechanism of small‐angle x‐ray scattering from the micro‐structures with a scale much smaller than the spatial resolution of the imaging system.^1^ As this technique can provide structural information that is inaccessible from the point of view of x‐ray absorption or x‐ray phase imaging,^3^ it offers new opportunities within a broad range of applications.^2^ In particular, dark‐field radiography is promising in the field of lung imaging, as the alveoli form fine tissue‐air structures that generate a considerable dark‐field signal but hardly contribute to the signal in the attenuation domain due to the large air fraction. After demonstrating the application of the method for lung imaging in animals,^4^ initial studies investigated the potential for human lung assessments.^5^, ^6^ The results from the following clinical studies in the context of lung diseases demonstrated that dark‐field chest x‐ray imaging allows the visualization and detection of COVID‐19‐pneumonia^7^ and the assessment and quantification of pulmonary emphysema in patients with chronic obstructive pulmonary disease (COPD).^8^, ^9^

However, these previous evaluations on the human lung utilized the analysis of the dark‐field signal in the inspiratory breathing state only. A recent study has now investigated the dark‐field signal characteristics in inspiration and expiration at the global lung level to discuss the influence of breathing state on the diagnostic value of the corresponding dark‐field radiography.^10^ Going beyond this evaluation and considering the substantial regional deformations of the lung during the respiratory cycle and the impact of diseases on the breathing dynamics, comparing different respiratory states on the local instead of a global level has the potential to expand the existing dark‐field lung signal evaluation approaches toward functional assessments.^4^ The challenge for such a comparative analysis is that a meaningful spatial correspondence between the images in the respiratory states is required, which must be addressed within the scope of a typical image registration task.

Image registration has evolved into an indispensable and widespread method in the clinical practice for accurately combining information from different image domains.^11^, ^12^ Because of the respiratory dynamics and variability, image registration is particularly important in lung imaging to reliably align data from different time points,^13^ patients,^14^ or modalities.^15^ The registered images can then be leveraged for diagnostics,^16^ disease progression monitoring,^17^ and treatment planning purposes.^18^ When it comes to the comparative analysis of lung image information throughout the respiratory cycle, image registration is a valuable tool specifically with regard to functional examinations.^13^, ^19^ For serial thoracic computed tomography (CT) scans, registration algorithms have been utilized within intensity‐based and Jacobian‐based methods that aim to provide an easily accessible and cost‐effective alternative for lung ventilation assessments to other standard techniques, such as positron emission tomography or Xenon‐enhanced CT.^20^ Moreover, a recent study proposed a novel hybrid method for estimating lung ventilation from CT scans by combining intensity and motion information.^21^ However, despite the wide application of thoracic CT image registration methods, it remains a very challenging task due to the vast three‐dimensional structural complexity and the large and anisotropic deformations.^22^ Furthermore, as a series of CT images is required for the above‐mentioned registration‐based ventilation analysis, the patient is exposed to a comparably high radiation dose. Dynamic chest radiography (DCR) aims to mitigate this issue by utilizing registered sequential plain chest radiographs instead of CT scans for functional examinations.^19^ Moreover, in contrast to standard CT, DCR can be performed in a sitting or standing patient position, reflecting physiologically relevant daily activity.^23^ Previous studies in the DCR context evaluated the utility of this approach in patients with various respiratory diseases such as COPD and COVID‐19.^24^, ^25^ To analyze pulmonary ventilation, DCR studies generally calculate X‐ray image pixel value change rates or differences.^26^, ^27^ However, to determine the local ventilation from the slight dynamic changes throughout the respiratory cycle, several additional image processing steps are necessary to separate the relevant variations from the influences of bone overlay in the lung area and blood flow changes.^23^

**FIGURE 1:**
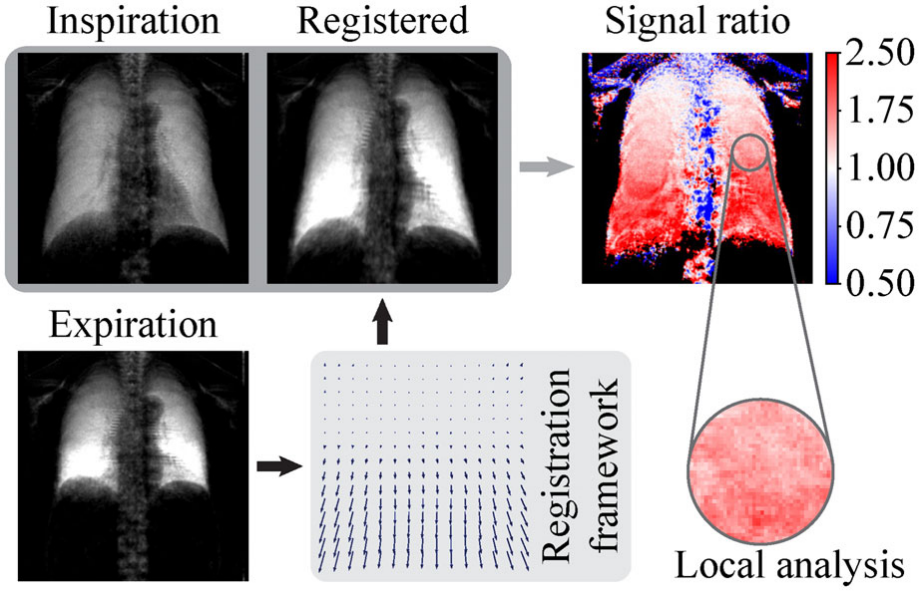
Dark‐field‐compatible deformable image registration methods enable matching chest radiographs in full expiration and full inspiration. The ratio of the transformed expiration and inspiration dark‐field images is used to analyze local lung signal changes.

In order to investigate the potential for extending the general DCR idea with dark‐field contrast and to expand the existing dark‐field radiography analysis approaches toward functional assessments, this article aims to discuss suitable image registration methods specifically for dark‐field chest radiographs to enable a local comparison of the lung signal in different phases of the respiratory cycle (see Figure 1). In addition, it is crucial for a comparative image analysis to employ suitable quantitative evaluation methods that can capture the characteristic dynamics of the signal changes properly. Inspired by the methodology used in DCR,^23^ we explore possible analysis approaches and discuss the differences between the dark‐field and commonly utilized attenuation signal domain.

In summary, the main contributions of our work are as follows:

1) We propose an iterative optimization‐based deformable registration framework that enables the matching of dark‐field chest radiographs acquired in different respiratory states. Since dark‐field chest radiography is still a relatively new imaging method compared to established techniques, the framework is the first to be specifically tailored for this registration task.
2) We investigate the performance of the proposed registration framework considering full inspiration and expiration dark‐field scans from a clinical COPD study and discuss registration evaluation metrics for this image‐matching task.
3) Considering the registered images, our local characterization of the lung dark‐field signal changes between respiratory states provides promising indicators that radiography‐based lung function assessment approaches may benefit from utilizing the dark‐field information in addition to the attenuation signal.

## 2 METHODS

### 2.1 Dark‐field chest radiography

**FIGURE 2:**
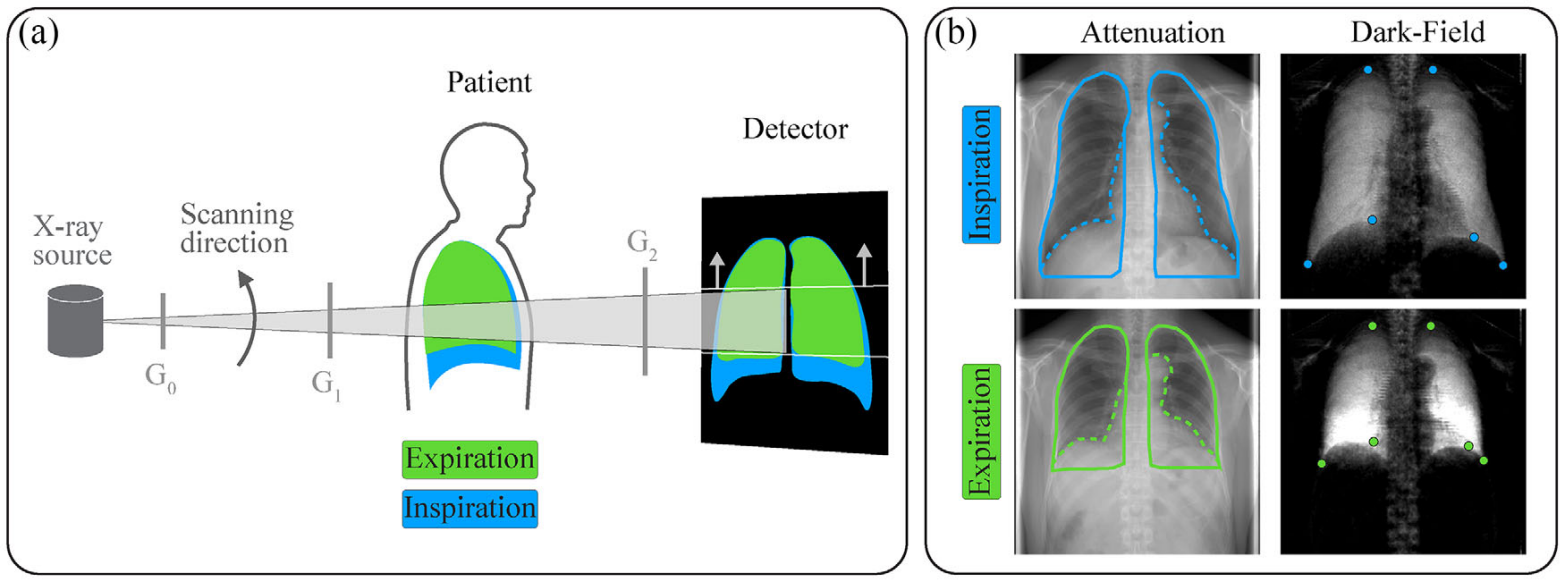
(a) Schematic view of the dark‐field chest x‐ray prototype system with the main components. The dark‐field lung image formation in the expiratory and inspiratory breath‐hold states is indicated in green and blue. (b) Examples of the corresponding attenuation and dark‐field images with an overlay of the manually drawn full (solid line) and partial (dotted line) lung masks and the annotated landmarks (blue and green dots).

The dark‐field radiographs studied in this article were acquired at a clinical dark‐field prototype system which is located at the university hospital Klinikum rechts der Isar, Technical University of Munich, Germany, and uses grating‐based x‐ray interferometry.^1^ A schematic view of the setup is shown in Figure 2a.

The Talbot‐Lau‐type interferometer consists of a source grating $G_{0}$ to provide spatially coherent x‐rays,^28^ the intensity pattern inducing grating $G_{1}$, and the analyzer grating $G_{2}$ in front of the detector (effective pixel size of 444 μm × 444 μm). For details regarding the x‐ray tube, gratings, and detector, refer to the work of Urban et al.^29^ As the fabrication of gratings large enough to cover the full desired field of view was not feasible at the time of construction, the radiography system works in scanning mode. This means that during image acquisition, the movable interferometer plane with the gratings scans the field of view from bottom to top within 6.5s (as indicated in Figure 2a).

Following the scanning procedure, the attenuation and dark‐field signals can be reconstructed simultaneously from the acquired data.^29^, ^30^, ^31^ Within this work, the dark‐field and attenuation signals were considered on a logarithmic scale, defined as D=−ln(v) with the visibility reduction v, and A=−ln(T) with the transmittance T.^32^ For the visualization in the figures of this work, a window range of 0.0 to 0.8 for the dark‐field signal and 0.0 to 8.0 for the attenuation signal was used, similar to our previous studies. Note that these ranges were only applied for visualization purposes and not in the course of the data processing and analysis presented here. As the utilized imaging method provides attenuation and dark‐field signals based on the same data recording, the two reconstructed images are perfectly co‐registered.

For our analyses, we used image data from a clinical study conducted among participants with or without COPD. The study was approved by the Institutional Ethics Committee of the Technical University of Munich and by the national Radiation Protection Agency (Z5–22462/2–2017‐021). All participants of this study gave written informed consent. Participants with signs of emphysematous impairment or healthy lungs, according to a CT evaluation, were included. Participants with pulmonary pathologies or conditions other than COPD were excluded from the study. For further details regarding the study, refer to the work of Willer et al.^8^ Within the study imaging protocol, attenuation and dark‐field images were acquired at maximum inspiration and full expiration in the lateral and posteroanterior (PA) orientations. PA image examples are depicted in Figure 2b. The dataset includes the respective images of a total of 95 participants.

### 2.2 Image registration

To establish the spatial correspondence between the PA attenuation or dark‐field images in the two respiratory states, we utilized image registration methods.

For the formal definition of the image registration task, we adopted the common description as an optimization problem between a fixed F and a moving image M that is to be transformed to the fixed image.^12^ Within this setting, the generic objective function for the transformation optimization consists of a similarity term $L_{\mathrm{sim}}$ and a regularization term $L_{\mathrm{reg}}$:

(1) $$L\left(\phi ,M,F\right)=L_{\mathrm{sim}}\left(M\;\circ \;\phi ,F\right)+\alpha L_{\mathrm{reg}}\left(\phi \right).$$

Within this objective function, the similarity term quantifies the level of alignment between the fixed image F and the transformed moving image M∘ϕ. The term $L_{\mathrm{reg}}$ regularizes the transformation ϕ and may be seen as a way to introduce prior knowledge regarding the transformation solution into the objective function.^12^ The regularization parameter α is used to adjust the relative influence of the regularization term to the similarity term within the objective function. Following this generic formulation, selecting the specific optimization method, objective function terms, and the deformation model for the transformation generally depends on the particular image registration task. The selections for our dark‐field chest radiograph registration framework will be introduced and explained in the following.

#### 2.2.1 Image preprocessing

Within the first step of the preprocessing, the study participant images were padded with zeros from their initial shape of 956 × 947 to 956 × 956 pixels. This simplifies the pixel distance calculations for downsampled and interpolated images, which is relevant for the evaluation metrics. Additionally, we manually corrected horizontal shifts between the inspiration and expiration scans for some study participants where a major lateral displacement was evident. The lateral shifts can occur if study participants reposition themselves within the setup cabin between the inspiration and expiration image acquisition. As the patients are placed with their chin on a support and their chest on a corresponding contact surface during the image acquisition, we assume that the corrections relate solely to lateral shifts, as only minor rotational movements and shifts along the beam direction are to be expected. Furthermore, for shorter computation times, the padded and shift‐corrected images were downsampled to 256 × 256 pixels, resulting in effective pixel dimensions of 1.66 × 1.66 mm.

#### 2.2.2 Radiograph registration

For the registration task, we defined the inspiration radiograph as the fixed (F) and the expiration scan as the moving image (M).

Due to the expected large and complex lung deformations between the full inspiratory and expiratory breathing state, we opted for a multi‐resolution strategy, which can handle local traps within the optimization space in such registration tasks.^33^ Within this approach, the downsampled input dark‐field images are further downsampled and smoothed to reduced complexity levels, and the degree of freedom of the transformation model is limited accordingly.

Regarding the non‐rigid transformation model for the dark‐field registration framework, we adopted a diffeomorphic spatial transformation approach using stationary velocity free‐form deformations (SVFFD).^34^ This approach was selected because the stationary velocity ansatz, by design, leads to consistent invertible transformations that preserve image topology, while the appealing aspects of the conventional free‐form deformation like its computational efficiency are retained.^35^

For the similarity term $L_{\mathrm{sim}}$ within the objective function for the dark‐field image registration task, we selected local normalized cross‐correlation (LNCC)^36^ over simpler and faster statistical similarity measures like the sum of squared differences (SSD) or the standard normalized cross‐correlation (NCC).

This is because the underlying assumption of SSD that the difference between the registered images should be zero except for noise,^37^ is not a sensible approach due to the potentially prominent dark‐field signal differences between the expiration and inspiration image (see e.g., Figure 2b) that should be preserved during the registration procedure. NCC is based on the more general assumption that there is a linear relationship between the two images on the global image domain scale.^37^ Extending the NCC assumption to a local image domain perspective, the LNCC similarity measure enables the registration framework to capture potentially different regional relationships between the dark‐field signal in distinct lung regions.

**FIGURE 3:**
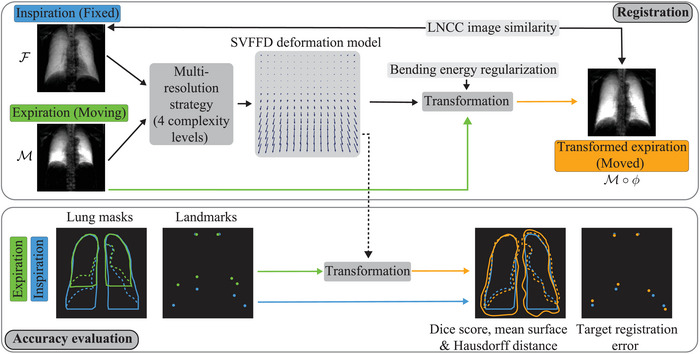
Overview of the dark‐field chest radiograph registration framework (top) and the accuracy evaluation approaches (bottom). The expiration dark‐field image is registered to the inspiration dark‐field radiograph utilizing a multi‐resolution strategy and an SVFFD deformation model. The LNCC image similarity between the inspiration and transformed expiration image as well as the bending energy regularization guide the transformation optimization. Manually annotated full and partial lung masks as well as landmarks are utilized for the registration accuracy evaluation. The registration transformation is applied to the respective masks and landmarks. The DICE, the MSD, the HD of the masks, and the TRE of the landmarks are calculated to assess the registration accuracy. DICE, Dice score; HD, Hausdorff distance; LNCC, local normalized cross‐correlation; MSD, mean surface distance; SVFFD, stationary velocity free‐form deformations; TRE, target registration error.

From an anatomical point of view, the movement of the lung tissue in the two‐dimensional projection plane should be continuous and without sliding or folding phenomena. Note that the sliding motion between rib cage and lung is irrelevant here since the rib cage does not generate a prominent dark‐field signal at the given prototype system setup conditions.^38^ This prior knowledge was included via a bending energy regularization term in the objective function to ensure the smoothness of the transformation. The bending energy is a popular penalty term for non‐affine transformations.^39^ As a compact overview, the combination of the selected components and the general workflow within the registration framework is depicted in the upper part of Figure 3.

To be able to compare the dark‐field image analysis with standard plain chest radiograph evaluations, we also implemented a framework for the registration of the corresponding attenuation images. For this purpose, we utilized a modified version of the dark‐field image registration framework described above.

As a first modification step, we opted for an additional affine pre‐registration procedure before the non‐rigid registration. This approach aimed to facilitate the local non‐rigid deformations with the increased information content of the attenuation images (bone/soft tissue signal in the lung region) by using an affine registration to establish a rough spatial correspondence in advance. The objective function for the affine pre‐registration procedure consists only of a masked NCC loss similarity term. The masking was necessary to enable a reasonable registration of the lung regions by reducing the impact of the collimator attenuation signal in some images and the extensive additional information content outside the lung region due to the bone and soft tissue structures. For the masking, we used the previously described partial lung mask for the inspiration image, dilated them by 25 pixels, and then computed the concave hull of the dilated masks (*concave_hull* Python package with concavity 300, see for example, the work from Park and Oh ^40^).

As a second modification step, we selected a masked LNCC loss within the non‐rigid multi‐resolution strategy transformation optimization for the same reasons as described in the context of the affine registration. The affinely pre‐registered expiration attenuation image was used as the moving image input for the non‐rigid registration procedure.

#### 2.2.3 Registration evaluation

As there is no ground truth transformation for our registration task, we used surrogate measures to assess the registration accuracy and regularity. For the accuracy evaluation, we utilized mask‐based and landmark‐based measures, see the lower part of Figure 3.

For the latter, a radiologist (co‐author A. W. Marka) with previous experience with dark‐field chest radiography annotated six landmarks to the inspiration and expiration dark‐field images, respectively, see Figure 2b. The annotated landmarks refer to points on the lung apex and on the costophrenic and costocardiac angles on the right and left sides of the lung. The respective landmarks were then transformed with the final registration transformation and the mean target registration error (TRE) was calculated.^11^ As the TRE is based on Euclidean distances between the landmarks, it has immediate physical meaning in how accurately the characteristic points in the lung match after registration.

Since the landmarks are sparsely distributed, we also assessed the accuracy via the mask‐based measures. The full and partial lung masks (see solid/dashed green and blue lines in Figure 2b) were manually drawn onto the attenuation images. Since the attenuation and dark‐field images are perfectly co‐registered within this imaging method, the masks generated using attenuation images also match the lung area in the corresponding dark‐field domain. The full masks include the heart region and the areas below the hemidiaphragm whereas the partial masks exclude these regions. For the mask‐based accuracy metrics, we chose the Dice score (DICE), symmetrical mean surface distance (MSD), and the symmetric Hausdorff distance (HD).^41^ DICE measures the overlap of the lung mask of the inspiration image and the transformed expiration lung mask whereas MSD and HD quantify the distance between the outlines of the masks. As the surface distance measures are based on Euclidean distances of mask surface points, they provide physical interpretability regarding how well the lung masks match after registration.

The transformation regularity, on the other hand, was evaluated based on the determinant of the Jacobian det(J)=det(∇ϕ).^41^ Similar to the work from Qiu et al.,^34^ we calculated the folding ratio, which is the fraction of pixels in the image domain where det(J)<0 (locations where the image topology is not preserved). In addition, we computed the mean magnitude of the gradient of the Jacobian determinant (MMGJD), which is an indicator of the spatial smoothness of the transformation.^34^

#### 2.2.4 Implementation details

To implement and test the previously described strategies and methods, we built upon the functionality of Deepali,^42^ a Python library that enables both iterative optimization‐based and learning‐based registration. Due to the limited data availability, only the iterative optimization functionalities and no learning‐based approaches were explored. Deepali provides a variety of different deformation models as well as similarity and regularization terms for the customization of a suitable objective function. Unless otherwise specified below, the corresponding functions and models from Deepali have been used within our registration frameworks. Deepali is built on the popular PyTorch machine‐learning library, allowing for fast registration optimization using graphics processing units (GPUs). This is also the main reason why we decided to use Deepali instead of other commonly used registration libraries that are still mainly reliant on CPU‐based optimization. In our case, all registration optimizations were conducted on *NVIDIA GeForce GTX 1080 Ti* GPUs.

Within the multi‐resolution strategy, we used four complexity levels corresponding to image dimension sizes of 256, 128, 64, and 32 pixels. A Gaussian kernel was used for smoothing with the default standard deviation of the respective function within Deepali. After the initialization of the four complexity levels, the transformation was optimized on the lowest‐resolution level first, until convergence (adaptive optimization) or the maximum number of optimization steps (3500 for the non‐rigid case) is reached. The convergence criterion within the adaptive optimization was that the absolute value of the difference between the current objective function value and the moving average within a window of the last 200 steps is lower than the defined convergence threshold ($10^{-3}$ times the initial objective function value). After the transformation optimization on a specific level, the same procedure was continued on the next complexity level until the input resolution was reached. The PyTorch Adam optimizer was used for all registration transformation optimizations.

The specific parameters used for the dark‐field and attenuation image registration algorithms are listed in Table 1.

**TABLE 1:** Registration Framework Parameters.

Parameter	Non‐rigid dark‐field	Affine attenuation	Non‐rigid attenuation
Stride (px)	(1, 8, 9, 10, 20)	—	10
LNCC kernel sizes (px)	11, 21, 41, 81	—	5, 11, 21, 41
Optimization steps	adaptive	800, 50, 50, 50	adaptive
Learning rate	$10^{-4}$	$10^{-3}$	$10^{-4}$
α	($1\cdot 10^{-3}$, 1, 100, 200, 500, $5\cdot 10^{5}$)	—	80

Overview of the selected parameters for the affine/non‐rigid registration implementations for the attenuation and dark‐field images. Listed numbers within brackets indicate varying parameters. Listed numbers without brackets indicate the parameter with increasing resolution level.

Abbreviation: LNCC, local normalized cross‐correlation.

As the non‐rigid transformations within the SVFFD approach are parameterized by cubic B‐spline functions,^39^ the distance between control points (stride) on the image pixel lattice needs to be provided for the transformation initialization. Since an appropriate stride‐regularization weighting factor α combination for the transformation model within the multi‐resolution approach is difficult to infer a priori, we evaluated the registration results for certain parameter combinations within sets of values (see Table 1). The same stride and α were used on all the resolution levels.

Furthermore, as the LNCC function calculates the NCC in local quadratic windows, suitable kernel sizes need to be provided for the registration parts that utilize LNCC (see Table 1). The specific kernel sizes for the dark‐field registration framework were selected qualitatively considering several typical inspiration dark‐field images within the dataset, so that the calculation window scale corresponds approximately to the width of one side of the lung in a dark‐field scan in the inspiratory breathing state at the corresponding resolution level. This aims to be able to capture different relationships on this scale while ensuring stability within the registration procedure. The same kernel sizes were used for all study participant image pairs within the dataset. For the non‐rigid attenuation image registration part, the kernel sizes were reduced to allow capturing different relations on smaller scales that may be caused by the overlapping bone and soft tissue structures in the lung area.

For the mask‐based accuracy assessments, the publicly available code implementations from the work of Qiu et al.^34^ were used to calculate the DICE, MSD, and HD.

### 2.3 Analysis of the registered images

In the context of dark‐field signal change quantification, we drew inspiration from earlier works that addressed analogous issues and followed a similar approach as in DCR. However, instead of the commonly used inter‐frame difference ansatz,^23^ we opted for an inter‐frame ratio approach. As the dark‐field signal can be expressed via the product of the dark‐field coefficient ε (representing the sample microstructure dependence) and the sample thickness d on the image pixel‐associated beam path as D=ε·d,^43^ we can form the ratio of the registered expiration and the inspiration dark‐field signal (for every image pixel):

(2) $$R_{D}=\frac{D_{\exp.,\mathrm{reg}.}}{D_{\mathrm{insp}.}}=\frac{\varepsilon_{\exp.,\mathrm{reg}.}}{\varepsilon_{\mathrm{insp}.}}\cdot \frac{d_{\exp.,\mathrm{reg}.}}{d_{\mathrm{insp}.}}.$$

In contrast to a signal difference ansatz, this ratio approach allows us theoretically to separate the relative dark‐field coefficient and lung thickness variation contributions to the dark‐field signal change (two fraction terms in Equation 2). The same evaluation method was used for the attenuation radiographs to compare both the attenuation ratio $R_{A}$ and the dark‐field ratio $R_{D}$ characteristics within the analyses.

We assessed the signal ratios with two approaches. Inspired by evaluations in DCR,^26^ we also projected the left and right lung signal ratios within the partial masks onto the craniocaudal (CC) axis. With this approach, we reduce the two‐dimensional signal ratio information to a simpler mean ratio graph which depends on the distance from the lung apex. The complexity is then further reduced to a scalar value by generating a linear fit for the graph to characterize signal ratio changes from upper to lower lung regions via the slope (CC gradient) of the fit. To fit a linear function to the mean signal ratio data, we utilized the *HuberRegressor* function from the Python *scikit‐learn* library. The *HuberRegressor* function was utilized as the linear regression loss function has the advantage of not being heavily influenced by outliers while not completely ignoring their effect. As the CC axis projections were conducted for the signal ratio within the left and right partial lung masks, the corresponding linear fits provide two CC gradient/slope values for every patient.

For the alternative dark‐field signal assessment approach, we divided the partial lung masks into an upper, middle, and lower region, see Figure 6, right. Then, we selected the signal ratio values within the respective region focus and calculated the mean dark‐field ratios in the three lung fields.

The evaluation results for the study participants were then further investigated concerning breathing capacity during imaging and COPD severity. The breathing capacity was quantified via the normalized relative vital lung capacity of the study participants: $VLC_{\mathrm{rel}}=\left(V_{\mathrm{insp}.}-V_{\exp.}\right)/V_{\mathrm{insp}.}$. For the $VLC_{\mathrm{rel}}$ calculation, the inspiration and expiration lung volumes $V_{\mathrm{insp}.}$, $V_{\exp.}$ were estimated utilizing the lateral and PA attenuation radiographs following the approach from Pierce et al.^44^

The relationship between CC gradients and mean signal ratios with the breathing capacity (represented by $VLC_{\mathrm{rel}}$) was then assessed using Spearman's rank correlation coefficient $r_{s}$ utilizing the *SciPy* Python package. In comparison to the Pearson product‐moment correlation coefficient, which aims to measure the degree of linearity of the variables, $r_{s}$ can capture the degree of monotonicity and is not limited to linear relationships.^45^ Furthermore, the Spearman correlation coefficient is more robust to outliers and regarding non‐normal data distributions.^45^ Given these characteristics, Spearman's rank correlation coefficient was selected to capture potential nonlinear relationships and non‐normal distributions of the variables in the context of the dark‐field signal within the quantitative analysis. A p‐value of less than 0.05 was considered to indicate a statistically significant relationship.

For the COPD severity quantification, we used Fleischner Society emphysema classification scores for the study participants, graded by trained radiologists after visual assessments of CT images, as described by Willer et al.^8^ Within this scheme, the study participants were divided into the following emphysema groups: 0 ‐ absent, 1 ‐ trace, 2 ‐ mild, 3 ‐ moderate, 4 ‐ confluent, and 5 ‐ advanced destructive.

## 3 RESULTS AND DISCUSSION

### 3.1 Registration of dark‐field radiographs

For the dark‐field registration experiments, we excluded the study participants where parts of the lung were out of the image domain, resulting in a total number of 87 image pairs.

#### 3.1.1 Regularization weighting and stride parameter impact

We explored the impact on the registration result within the α‐stride parameter space via multiple evaluation metrics. The impact on the medians of the DICE and the MSD for the full and partial lung masks, as well as the influence on the TRE, MMGJD, and the registration duration, are summarized in Table 2. Median values were evaluated to capture the behavior of potentially skewed distributions with outliers.

**TABLE 2:** Framework Parameter Registration Impact.

α, Stride	DICE full	DICE part.	MSD full (mm)	MSD part. (mm)	TRE (mm)	MMGJD $\cdot 10^{-3}$	Duration (s)
$1\cdot 10^{-3}$, 1	0.914	0.818	5.89	10.15	13.64	710	338
1, 1	0.921	0.847	5.63	8.78	12.97	406	243
$1\cdot 10^{-3}$, 8	0.937	0.932	4.70	3.79	7.38	172	332
1, 8	0.943	0.936	4.33	3.57	5.97	37	157
1, 9	0.944	0.937	4.27	3.49	5.81	28	159
1, 10	0.944	0.937	4.12	3.45	5.87	23	156
100, 8	0.949	0.937	3.81	3.46	5.85	6	105
100, 9	0.951	0.936	3.78	3.49	5.95	5	127
100, 10	0.950	0.936	3.71	3.52	6.10	4	103
200, 8	0.950	0.936	3.74	3.49	6.04	4	99
200, 9	0.951	0.936	3.72	3.51	6.26	4	118
200, 10	0.951	0.935	3.69	3.59	6.36	3	101
500, 8	0.951	0.935	3.68	3.58	6.32	3	104
500, 9	0.952	0.934	3.65	3.73	6.55	3	121
500, 10	0.952	0.933	3.62	3.81	6.80	2	112
500, 20	0.948	0.920	3.97	4.64	7.98	0.7	113
$5\cdot 10^{5}$, 10	0.858	0.828	11.42	10.30	30.36	0.3	75
$5\cdot 10^{5}$, 20	0.851	0.820	11.79	10.64	31.03	0.3	59

Impact of the regularization parameter α and the transformation model stride on the median of evaluation metrics and the optimization duration.

Abbreviations: DICE, Dice score; full, full lung masks; MMGJD, mean magnitude of the gradient of the Jacobian determinant; MSD, mean surface distance; part., partial lung masks; TRE, target registration error.

The median DICE for the full lung mask shows improved values for increasing regularization weighting and stride up until the (α=500 or $5\cdot 10^{5}$; stride = 10 or 20) combinations. The median DICE for the partial lung mask exhibits the opposite trend after an initial increase throughout the ($\alpha =1\cdot 10^{-3}$ or 1; stride = 1 or 8) combinations. The median MSD for the full lung mask shows better values for increasing regularization strength and stride up until the (α=500 or $5\cdot 10^{5}$; stride = 10 or 20) combinations. The partial mask median MSD first improves for increasing regularization weighting and stride but then deteriorates after α=100. The same holds for the median TRE. Within the explored parameter space, regularization weighting of $\alpha =1\cdot 10^{-3}$ leads to the highest median MMGJD values and registration durations. This behavior is expected as the bending energy loss imposes fewer restrictions and more less‐smooth transformation states are accessible in the optimization search space for weak regularization. In all the ($\alpha =1\cdot 10^{-3}$; stride = 1) combination cases, this also led to considerable median folding ratios in the range of $5.80\cdot 10^{-4}$ up to 0.13. Furthermore, for the configuration (α=1, stride = 8), a nonzero folding ratio in the order of $10^{-4}$ was still observed for two cases. This indicates that such low regularization strengths or stride values lead to violations of topology preservation, even for the SVFFD model, where we would not expect folding due to the velocity field formulation.

Overall, we observe a strong deterioration in all evaluation metrics in the extremal explored α and stride cases, which illustrate the excessive transformation freedom scenario ($\alpha =1\cdot 10^{-3}$; stride = 1 combinations) and the extreme transformation restriction scenario ($\alpha =5\cdot 10^{5}$; stride = 20 combinations). In the remaining more moderate parameter combination scenarios (α= 1, 100, 200, 500; stride = 8, 9, 10), the changes in the median evaluation metrics are relatively small, except for the MMGJD and registration duration. This is because the effective pixel dimensions for the downsampled input images are 1.66 mm, whereas the magnitude of MSD and TRE variations is in the order of 1 mm in this moderate parameter combination range. Moreover, if we consider the metric behaviors described above, the opposing trends within the moderate parameter combination range make it impossible to select the optimal configuration within the explored search space. Only the image domain folding and less smooth transformations for α=1 and stride = 8 revealed that this parameter configuration within the moderate combination range is not suitable for our task. Apart from that, the configurations within the moderate α‐stride range generally provide high Dice and low surface distance and TRE scores, considering that uncertainties due to the manual annotation of the lung masks and landmarks are to be expected. Considering all findings, we selected the model configuration (α=100, stride = 10) for further registration experiments.

#### 3.1.2 Selected configuration performance details

**FIGURE 4:**
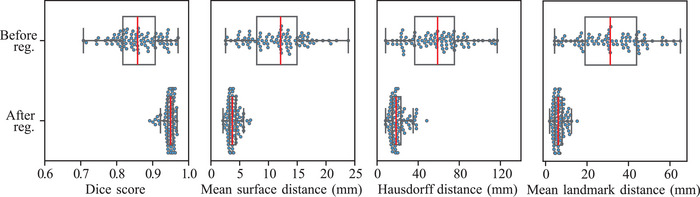
Registration evaluation metrics considering full lung mask metrics and landmark distances (TRE) for the dark‐field image registration framework within the dataset. The top row shows the scores before, and the bottom row the values after the registration procedure. Red lines indicate the median, black boxes the interquartile range (25th to 75th percentile), and blue dots the individual study participant scores. TRE, target registration error.

Based on the selected parameter configuration, the performance of the registration framework on the dataset was investigated in more detail. Figure 4 shows a comparison of the full mask metric score and TRE distributions before (upper row) and after the registration procedure (bottom row). The corresponding partial mask metric score distributions are depicted in Figure S1 of the Supporting Information material.

Considering the distribution medians, we observed a pronounced increase in the Dice score from 0.858 to 0.950, an MSD reduction from 12.1 to 3.7 mm, and a substantial drop in the HD from 58.9 to 18.5 mm. Similar behavior was observed for the partial masks with median DICE shifting from 0.835 to 0.936, a median MSD reduction from 10.4 to 3.5 mm, and an HD decrease from 63.1 to 18.9 mm. The analogous response of the full and partial mask‐based metrics is not surprising, as they are identical in many regions. Nevertheless, the assessment with the full and partial masks is important to inspect the behavior of the registration below and at the diaphragmatic arches as well as at the borders to the heart area. A substantial improvement of the median from 31.2 to 6.1 mm is also recognizable for the mean distances of the landmarks (TRE). The landmark evaluation approach has the advantage over the surface distance metrics that the performance can be examined at specific characteristic points of the lung in the dark‐field domain instead of an entire lung region surface. One general limitation of the metrics used, however, is that the landmarks and masks utilized can be used to evaluate the accuracy of registration mainly at the boundaries of the lungs to the thorax, diaphragm, or heart, but not at central points within the lung.

**FIGURE 5:**
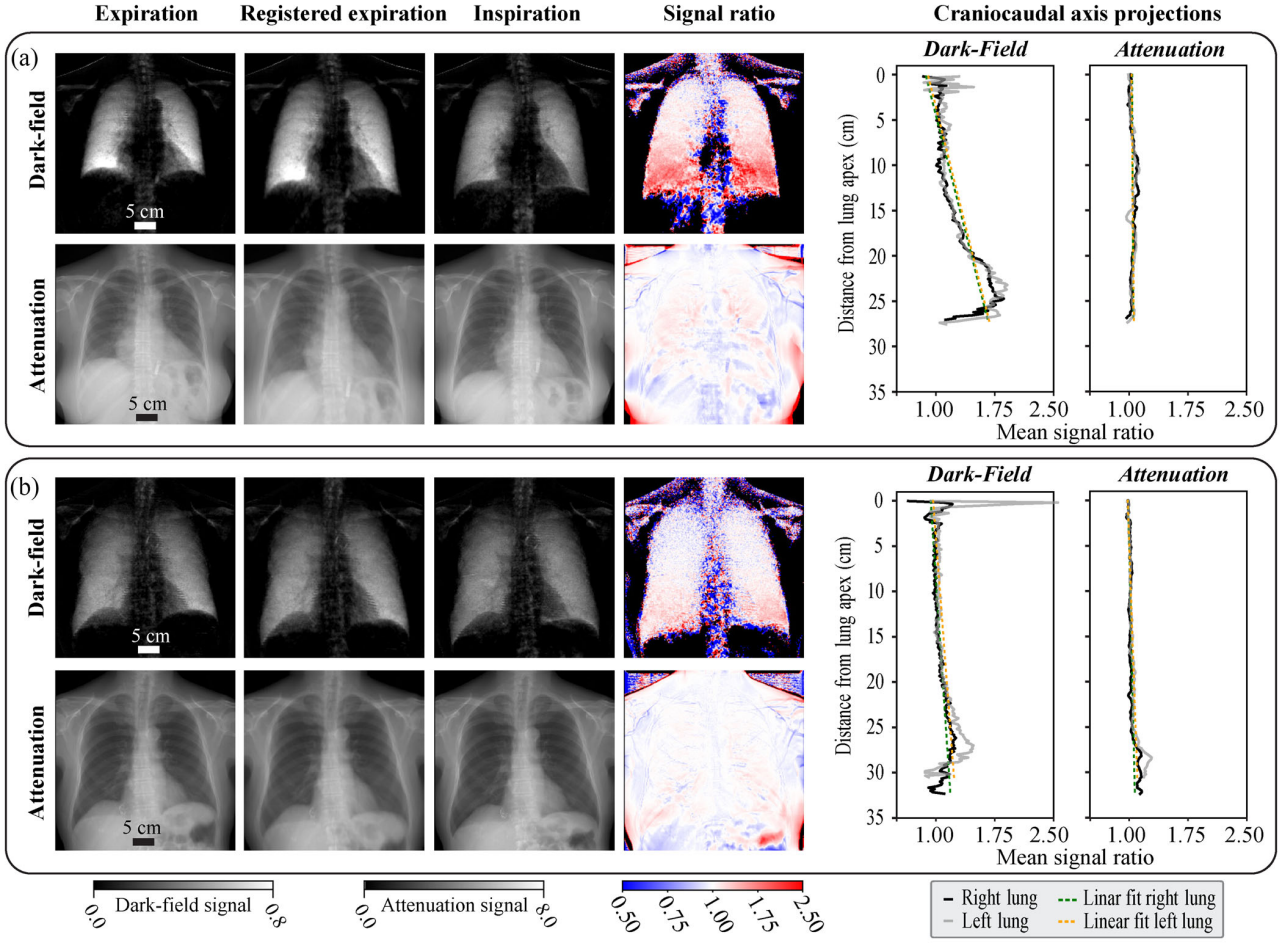
Attenuation and dark‐field image registration results for two study participants with signal ratio analysis. (a) Fleischner scale score 0, $VLC_{\mathrm{rel}}=0.28$. (b) Fleischner scale score 2, $VLC_{\mathrm{rel}}=0.14$. The linear fit slopes to the CC signal ratio projection differ substantially for the two study participant cases in the dark‐field domain (0.029 right and left for case (a), 0.008 right and 0.009 left for case (b)) in contrast to the attenuation domain (0.001 right and left for case (a), 0.003 right and 0.004 left for case (b)). Black regions within the ratio images indicate pixels where the transformed expiration or inspiration signal was below $10^{-15}$ and thus set to *NaN* to avoid division by zero errors.

Overall, despite the considerable variance of the initial configuration of the lung in expiration and inspiration (broad distributions in the top row of Figure 4), the registration framework demonstrates consistently good performance for all cases (narrow distributions in the bottom row of Figure 4). In line with the quantitative evaluation metrics, the visual qualitative evaluation confirmed the good performance of our dark‐field registration framework within the dataset (see e.g., Figure 5a,b).

### 3.2 Analysis of the registered images

The successfully registered images were then utilized for the evaluation of the transformed expiration‐inspiration dark‐field signal ratios. Two examples of the dark‐field ratio $R_{D}$ evaluations are depicted alongside the initial and registered image in the top row of Figure 5a,b. Structures other than the lung, such as the clavicle bones or the spine, are also visible within the signal ratio images. However, the focus is on the signal ratio in the lungs, which is why the quantitative analyses were carried out using lung masks.

#### 3.2.1 Dark‐field and attenuation domain comparison

To illustrate the differences between the signal change in the lung from inspiration to expiration in the dark‐field and attenuation domains, the registered attenuation images with the corresponding signal ratios $R_{A}$ are displayed in the bottom row of Figure 5a,b. These two study participants exemplify differences in signal changes primarily related to the breathing capacity during the image acquisition. The study participant in Figure 5a represents the higher breathing capacity cases, indicated by $VLC_{\mathrm{rel}}=0.28$. In contrast, the study participant in Figure 5b represents the lower breathing capacity cases, indicated by $VLC_{\mathrm{rel}}=0.14$.

Comparing the two signal domains, more distinct signal ratio differences between the cases are recognizable in the dark‐field than in the attenuation domain. This is partly because the magnitude of the changes in the lung area is larger in the dark‐field signal. But more importantly, there is signal overlap from different objects in the attenuation domain. In particular, streaks are recognizable in the lung area of the attenuation signal ratio in many areas caused by the clavicles and ribs. As these bone structures do not follow the movement of the lung tissue, the registration algorithm for the attenuation domain is unable to correctly map these structures to each other when correcting for lung tissue motion. As these bone structures only generate a very weak dark‐field signal compared to lung tissue, this issue is overcome to a large extent with the registered dark‐field radiographs. In the attenuation domain, this could only be solved by additional image processing steps such as bone suppression algorithms. The same signal overlap aspect also applies to non‐lung soft tissue structures such as breast fat tissue, which generates a substantial attenuation signal and can superimpose the lung area in considerably different locations in the expiratory and inspiratory state. The bone and soft tissue contributions to the signal just described also have corresponding consequences for the complexity of the lung registration task for the algorithm in the attenuation domain compared to the dark‐field domain. This is reflected accordingly in the additional measures, such as affine pre‐registration and masking steps in the dedicated implementation of the attenuation image registration algorithm. This further highlights the reduced complexity of the lung registration problem in the dark‐field domain by focusing on the lung tissue and thus the opportunities with regard to lung dynamics analysis with registered dark‐field radiographs. Accordingly, it could also be interesting for future studies to apply the image transformation found in the dark‐field domain to the (perfectly co‐registered) attenuation counterparts in order to achieve improved registration of the lung region in the attenuation images. However, it is important to note here that, for example, the lack of information about bone and tissue information in the vicinity of the lung or extreme signal loss in particularly strong emphysema regions in the dark‐field domain could lead to potential mis‐registration phenomena during transformation transfer to the attenuation domain. Therefore, this transformation domain transfer would first have to be thoroughly examined qualitatively and quantitatively before it could potentially be leveraged in future studies, which is not within the scope of this work.

To quantitatively assess the differences in signal ratios between the dark‐field and attenuation domains, we evaluated the CC axis gradients, see right side of Figure 5a,b. For the example cases, prominent slope differences are observed within the dark‐field domain in contrast to the attenuation image domain. This indicates that signal change differences connected with breathing capacity can be analyzed more easily and more accurately by comparing the dark‐field signal in different respiratory states. To substantiate this hypothesis derived from the single study participant pair, we compared the CC axis gradient about $VLC_{\mathrm{rel}}$ for 53 study participants (attenuation registration metric medians: full/part. mask DICE 0.943/0.924, MSD 3.82/3.91 mm, HD 19.90/20.24 mm). The other study participants were excluded from this evaluation due to insufficient attenuation image registration quality. We found stronger correlations of the dark‐field‐based slopes (Spearman corr. coeff.: $r_{s,right}=0.55$, p<0.01 and $r_{s,left}=0.48$, p<0.01) compared to the attenuation‐based CC gradients (Spearman corr. coeff.: $r_{s,right}=0.20$, p=0.16 and $r_{s,left}=0.40$, p<0.01). The correlation of the attenuation‐based CC slope on the right side of the lung is not even significant, considering the utilized significance threshold of p=0.05. In addition to the stronger correlations, the dark‐field‐based slopes change over a broader range (dark‐field: ≈−0.02 to 0.06, attenuation: ≈0.00 to 0.02). Combined, this indicates a general trend that the CC gradient in the dark‐field domain shows more pronounced changes with the breathing capacity than in the attenuation domain. In comparison to other functional lung imaging approaches, there are also potential opportunities in this observation with regard to lung signal change sensitivity in terms of dose exposure for the subjects examined. Although the determined effective radiation dose for a postero‐anterior scan in the dark‐field prototype system of 0.035 mSv^8^ is considerably lower than the typical radiation dose of 1‐10 mSv in routine CT examinations,^46^, ^47^ it is comparable to the average effective dose of 0.02 mSv for a postero‐anterior scan in standard chest radiography setups^48^ (at increased information content, obtaining dark‐field and attenuation information simultaneously). Moreover, investigations have shown that the dose in the current dark‐field radiography prototype system can be further reduced while maintaining the same diagnostic value.^49^ Thus, our functional lung imaging approach holds the prospect of reducing the radiation dose for patients compared to the standard DCR, as the observed lung signal change sensitivity advantages in the dark‐field domain promise to enable a reduction in the number of images required for the examination.

#### 3.2.2 Lung field signal characterization

**FIGURE 6:**
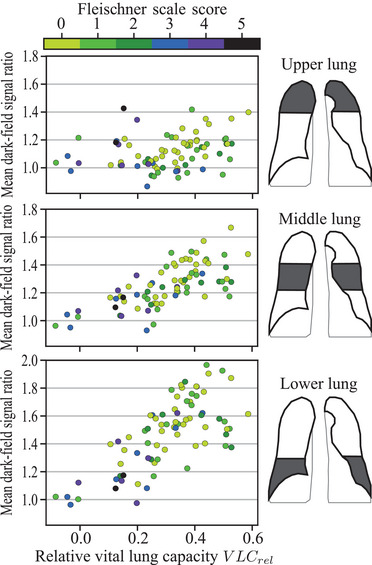
Mean dark‐field signal ratio in the three lung regions in relation to $VLC_{\mathrm{rel}}$. Spearman correlations: $r_{s,\mathrm{upper}}=0.30$ (p=0.01), $r_{s,\mathrm{middle}}=0.71$ (p<0.01), $r_{s,\mathrm{lower}}=0.67$ (p<0.01). The color map indicates the Fleischner scale score of the study participants, representing the COPD (emphysema) severity (0 ‐ absent, 1 ‐ trace, 2 ‐ mild, 3 ‐ moderate, 4 ‐ confluent, and 5 ‐ advanced destructive).

Since in some cases, a nonlinear course was observed in the CC dark‐field signal ratio projection graphs, see for example, Figure 5a, which a linear fit cannot capture adequately, we evaluated an alternative approach to characterize the regional dark‐field signal changes. This is visualized in Figure 6, where we calculated the mean dark‐field signal ratio in the upper, middle, and lower lung regions and evaluated these concerning $VLC_{\mathrm{rel}}$ for 77 study participants. The other study participants were excluded from this evaluation due to missing CT scans, out‐of‐image lung regions, or non‐standard emphysema type (lymphangioleiomyomatosis, pulmonary fibrosis).

In general, different characteristics of the dark‐field signal change in connection with $VLC_{\mathrm{rel}}$ were found in the three lung fields. There is a weak correlation ($r_{s,\mathrm{upper}}=0.30$, p=0.01) of the mean dark‐field signal ratio within the upper lung region, but strong correlations within the middle ($r_{s,\mathrm{middle}}=0.71$, p<0.01) and lower ($r_{s,\mathrm{lower}}=0.67$, p<0.01) lung region. In addition to the stronger correlations, the ratio changes over an increasingly broader range in the middle and lower lung fields, respectively.

Combined, the observed characteristics in connection with $VLC_{\mathrm{rel}}$ indicate that this analysis approach allows to capture the different relative alveolar density changes between expiration and inspiration in the distinct lung fields. This is because we expect increasing local lung volume changes in the CC direction due to the dominant motion of the diaphragm during the breathing cycle. The rationale behind this is that we expect an increase in the dark‐field signal during exhalation in contracting lung areas due to increased X‐ray scattering as a result of the larger number of tissue‐air interfaces along the beam path when the alveolar density is increased in these regions. This then leads to a corresponding increase in the local dark‐field signal ratio proportional to the dark‐field coefficient expiration‐inspiration ratio in Equation 2, as the dark‐field coefficient ε is expected to be primarily dependent on the tissue microstructure introduced by the pulmonary alveoli in the lung case. Considering that the gas exchange takes place at the alveoli, the observed local changes in the dark‐field signal are in good agreement with regional lung ventilation findings in single photon emission tomography (SPECT) in upright standing subjects.^50^ Hence, our proposed dark‐field signal‐based functional analysis approach is promising for a radiography‐based regional lung ventilation assessment via local alveolar density change mapping. In the clinical context, the new information provided by the comparison of the lung dark‐field signal between registered images from different respiratory states opens up new opportunities for an imaging‐based regional lung ventilation assessment with a simple and low‐dose acquisition procedure. In view of the current situation in clinical practice, where lung ventilation is predominantly evaluated globally using pulmonary function tests and local, imaging‐based assessment approaches are often complex and therefore unsuitable for application in large patient populations and longitudinal monitoring, there is an urgent need for the development of new methods in this regard in the future. To be able to better situate the proposed dark‐field radiography‐based approach within the context of other lung ventilation imaging approaches, it would be appropriate to conduct further studies in the future in which the respective study participants are also examined with other functional imaging techniques like SPECT to enable a patient‐specific quantitative comparison.

Moreover, from the quantitative analysis perspective, it should be noted as a limitation that the complex three‐dimensional lung movement is considered here in the two‐dimensional projection. As with the corresponding registration methods in DCR, the issue is that the dark‐field registration algorithm is not capable of correctly capturing the deformation of the lung tissue along the beam direction due to the lack of additional spatial information. The resulting influence of the deformation along the beam direction on the quantitative analysis using the dark‐field signal ratio is also evident via the ratio term between the local lung thicknesses in the two breathing states in Equation 2. To be able to focus on the relative change in the dark‐field coefficient and thus on the relative change in alveolar density in the quantitative evaluation of the signal ratio, information on the local change in lung thickness along the beam direction would have to be integrated. To mitigate this limitation in future studies, currently investigated methods for predicting the local lung thickness on conventional 2D radiographs^51^ could be utilized to capture the lung deformation along the beam direction in the quantitative analysis.

To evaluate the relationship between $VLC_{\mathrm{rel}}$, signal ratio, and COPD severity, the Fleischner scale score is color‐coded within Figure 6. This way, it can be observed that the more severe emphysema cases are clustered at small $VLC_{\mathrm{rel}}$ and correspondingly low dark‐field signal ratios. This was to be expected, as emphysematous lung tissue restricts the flexibility of the lungs, accordingly leading to breathing impairments. However, data points from low Fleischner score study participants with $VLC_{\mathrm{rel}}$ close to zero and data instances from study participants with more severe emphysema with greater $VLC_{\mathrm{rel}}$ can also be observed. Moreover, it can be presumed that the study participants with $VLC_{\mathrm{rel}}<0$ did not correctly follow or understand the exhale‐inhale breathing instructions as such values are implausible. It is therefore important to note that it is crucial how well the study participant follows the breathing instructions during the image recordings. This, in turn, complicates the analysis of specific relationships between the dark‐field signal change and the lung disease severity. However, it should be noted here that this problem of uncertainty as to whether the respective images were indeed acquired in the intended respiratory state also exists with other dynamic lung imaging approaches such as 4DCT or DCR, which means that outliers are difficult to avoid without increased effort in general. In our specific dark‐field chest radiography workflow, one approach would be to practice the breathing procedure during imaging with the patient once before the actual acquisition to ensure that the instructions are understood. In addition, one could potentially resort to in situ pulmonary function tests during imaging in future studies if more precise information about the actual breathing behavior is required.

Yet, it may be already of interest for further studies to evaluate local mean dark‐field signal ratio differences with respect to $VLC_{\mathrm{rel}}$ and the Fleischner scale score in the current dataset, to explore the possibility of differentiating disease severity classes of study participants via the dark‐field signal changes between breathing states. This would then be a further step toward investigating the potential of the presented dark‐field radiography‐based functional assessment approach with regard to a supportive role in early detection and long‐term monitoring of obstructive lung diseases such as COPD. In COPD cases with very early‐stage emphysema, this aims to facilitate the identification of pathological patterns via dark‐field signal alterations in the lung at an early stage, even before structural changes can be detected on conventional radiographs or CT scans. Moreover, given that chest radiography is a widely used evaluation method in everyday clinical practice and that the dark‐field radiography setup provides lung tissue‐specific dark‐field signal information in addition to the attenuation contrast used to date, this makes the presented approach interesting for routine clinical application in the context of low‐dose early detection and long‐term monitoring of lung diseases. To explore the broad application potential in this context in more detail, further studies with more participants and other lung diseases need to be evaluated in the future to improve the statistical significance of the current evaluations and to verify the applicability to various lung conditions. In this regard, further participants with and without COPD are still being recruited within the clinical study described above, which will allow us to include more data regarding the functional dark‐field lung analysis in future studies and to also examine more subjects within the higher Fleischner scale score classes.

## 4 CONCLUSION

In this study, we presented a framework specifically tailored for the registration of dark‐field chest radiographs acquired in different breathing states. Apart from the scenarios with insufficient or excessive transformation regularization and very low/high stride selection, the examined parameter configurations performed well in terms of the evaluation metrics used. The selected framework configuration allows establishing an adequate spatial correspondence between dark‐field chest radiographs in different respiratory states and thus to perform a local signal change analysis. This allows us to add a dynamic comparative dimension to the previous static analysis approaches of the dark‐field lung signal that considered one breathing state only.

In addition to the benefits regarding bone and soft‐tissue overlap contributions in the lung region, our comparison between the dark‐field and attenuation domains using the gradient of the CC axis projections of the registered image signal ratios presented evidence of slope‐sensitivity advantages concerning $VLC_{\mathrm{rel}}$ in the dark‐field domain. This indicates that radiography‐based lung function assessment approaches can benefit from the utilization of lung signal information in the dark‐field domain in addition to the commonly used attenuation images. The enhanced lung sensitivity may also help to reduce the number of images that need to be acquired to be able to analyze the signal changes concerning a local lung function estimation and thus to further reduce the radiation dose for patients compared to the standard DCR. In a clinical context, this could make the proposed approach particularly interesting for use across a broad patient spectrum for the early detection of lung pathologies and the long‐term monitoring of disease progression from a functional perspective.

Our alternative analysis approach of examining the mean signal ratio in the three lung fields provided insight into the distinct behavior of the dark‐field signal changes with the breathing capacity. This enabled the respiratory state‐related changes in the lung dark‐field signal to be characterized for the first time on a local level using actual clinical data. This is an important contribution to a better understanding of local signal dependencies for the further development of the relatively new dark‐field lung imaging method, but also with regard to the influence on the diagnostic value in the clinical context. Moreover, the local signal characteristics are in good agreement with the expected lung volume changes in the respective lung regions, indicating that our analysis approach is promising for investigating local alveolar density changes during the breathing cycle. This opens up new options for low‐dose and rapid lung ventilation assessment via dark‐field chest radiography that could considerably improve lung diagnostics in terms of early detection of pathologies and disease progression monitoring from a functional perspective.

## CONFLICT OF INTEREST STATEMENT

Thomas Koehler is an employee of Philips Innovative Technologies. The other authors declare no conflicts of interest.

## Supporting information

Supporting Information

## References

[1] Pfeiffer F , Bech M , Bunk O , et al. Hard‐X‐ray dark‐field imaging using a grating interferometer. Nat Mater. 2008;7(2):134‐137. doi:10.1038/nmat2096 18204454

[2] Yashiro W , Terui Y , Kawabata K , Momose A . On the origin of visibility contrast in x‐ray Talbot interferometry. Opt Express. 2010;18(16):16890‐16901. doi:10.1364/OE.18.016890 20721081

[3] Fitzgerald R . Phase‐sensitive x‐ray imaging. Phys Today. 2000;53(7):23‐26. doi:10.1063/1.1292471

[4] Gradl R , Morgan KS , Dierolf M , et al. Dynamic in vivo chest x‐ray dark‐field imaging in mice. IEEE Trans Med Imaging. 2018;38(2):649‐656. doi:10.1109/TMI.2018.2868999 30188818

[5] Fingerle AA , De Marco F , Andrejewski J , et al. Imaging features in post‐mortem x‐ray dark‐field chest radiographs and correlation with conventional x‐ray and CT. Eur Radiol Exp. 2019;3(1):25. doi:10.1186/s41747-019-0104-7 31292790 PMC6620231

[6] Gassert FT , Urban T , Frank M , et al. X‐ray dark‐field chest imaging: qualitative and quantitative results in healthy humans. Radiology. 2021;301(2):389‐395. doi:10.1148/radiol.2021210963 34427464

[7] Frank M , Gassert FT , Urban T , et al. Dark‐field chest X‐ray imaging for the assessment of COVID‐19‐pneumonia. Commun Med (Lond). 2022;2(1):147. doi:10.1038/s43856-022-00215-3 36411311 PMC9678896

[8] Willer K , Fingerle AA , Noichl W , et al. X‐ray dark‐field chest imaging for detection and quantification of emphysema in patients with chronic obstructive pulmonary disease: a diagnostic accuracy study. Lancet Digit Health. 2021;3(11):e733‐e744. doi:10.1016/S2589-7500(21)00146-1 34711378 PMC8565798

[9] Urban T , Sauter AP , Frank M , et al. Dark‐field chest radiography outperforms conventional chest radiography for the diagnosis and staging of pulmonary emphysema. Invest Radiol. 2023;58(11):775‐781. doi:10.1097/RLI.0000000000000989 37276130 PMC10581407

[10] Urban T , Gassert FT , Frank M , et al. Dark‐field chest radiography signal characteristics in inspiration and expiration in healthy and emphysematous subjects. Eur Radiol Exp. 2025;9(1):40. doi:10.1186/s41747-025-00578-x 40146395 PMC11950489

[11] Hill DLG , Batchelor PG , Holden M , Hawkes DJ . Medical image registration. Phys Med Biol. 2001;46(3):R1. doi:10.1088/0031-9155/46/3/201 11277237

[12] Sotiras A , Davatzikos C , Paragios N . Deformable medical image registration: A survey. IEEE Trans Med Imaging. 2013;32(7):1153‐1190. doi:10.1109/TMI.2013.2265603 23739795 PMC3745275

[13] Murphy K , Pluim JPW , Van Rikxoort EM , et al. Toward automatic regional analysis of pulmonary function using inspiration and expiration thoracic CT. Med Phys. 2012;39(3):1650‐1662. doi:10.1118/1.3687891 22380397

[14] Li B , Christensen GE , Hoffman EA , McLennan G , Reinhardt JM . Establishing a normative atlas of the human lung: intersubject warping and registration of volumetric CT images. Acad Radiol. 2003;10(3):255‐265. doi:10.1016/S1076-6332(03)80099-5 12643552

[15] Pan H , Zhou C , Zhu Q , Zheng D . A fast registration from 3D CT images to 2D X‐ray images. In: *2018 IEEE 3rd International Conference on Big Data Analytics (ICBDA)*. Shanghai, China. IEEE; 2018:351‐355. doi:10.1109/ICBDA.2018.8367706

[16] Li M , Castillo E , Luo HY , et al. Deformable image registration for temporal subtraction of chest radiographs. Int J Comput Assist Radiol Surg. 2014;9(4):513‐522. doi:10.1007/s11548-013-0947-y 24078349

[17] Stavropoulou A , Szmul A , Chandy E , Veiga C , Landau D , McClelland JR . A multichannel feature‐based approach for longitudinal lung CT registration in the presence of radiation‐induced lung damage. Phys Med Biol. 2021;66(17):175020. doi:10.1088/1361-6560/ac1b1d PMC8395598 34352743

[18] Lei Y , Fu Y , Wang TX , et al. 4D‐CT deformable image registration using multiscale unsupervised deep learning. Phys Med Biol. 2020;65(8):085003. doi:10.1088/1361-6560/ab79c4 32097902 PMC7775640

[19] Fyles F , FitzMaurice TS , Robinson RE , Bedi R , Burhan H , Walshaw MJ . Dynamic chest radiography: a state‐of‐the‐art review. Insights Imaging. 2023;14(1):107. doi:10.1186/s13244-023-01451-4 37332064 PMC10277270

[20] Ding K , Cao K , Amelon RE , Christensen GE , Raghavan ML , Reinhardt JM . Comparison of intensity‐ and Jacobian‐based estimates of lung regional ventilation. In: Third International Workshop on Pulmonary Image Analysis. lungworkshop; 2010:49‐60.

[21] Tzitzimpasis P , Ries M , Raaymakers BW , Zachiu C . Hybrid method for estimating lung ventilation from CT by combining intensity and motion information. Med Phys. 2025;52(6):4528‐4539. doi:10.1002/mp.17787 40159526 PMC12149685

[22] Xiao H , Xue X , Jiang X , et al. Deep learning‐based lung image registration: a review. Comput Biol Med. 2023;165:107434. doi:10.1016/j.compbiomed.2023.107434 37696177

[23] Hata A , Yamada Y , Tanaka R , et al. Dynamic chest X‐ray using a flat‐panel detector system: Technique and applications. Korean J Radiol. 2021;22(4):634‐651. doi:10.3348/kjr.2020.1136 33289365 PMC8005348

[24] Abe T , Yoshida N , Shimada T , Nakashima M , Nagai A . Respiratory frequency‐tunable dynamic imaging for lung function: new exam method using chest X‐ray cine imaging considering various respiratory diseases. PLoS One. 2022;17(11):e0276859. doi:10.1371/journal.pone.0276859 36395105 PMC9671319

[25] Robinson RE , Fyles F , Burton RC , et al. The utility of dynamic chest radiography in patients with asthma, COPD, COVID‐19 and ILD: a pilot study. Pulmonology. 2025;31(1):2436274. doi:10.1080/25310429.2024.2436274 39925252

[26] Yamada Y , Ueyama M , Abe T , et al. Difference in the craniocaudal gradient of the maximum pixel value change rate between chronic obstructive pulmonary disease patients and normal subjects using sub‐mGy dynamic chest radiography with a flat panel detector system. Eur J Radiol. 2017;92:37‐44. doi:10.1016/j.ejrad.2017.04.016 28624018

[27] Tanaka R , Sanada S , Okazaki N , et al. Detectability of regional lung ventilation with flat‐panel detector‐based dynamic radiography. J Digit Imaging. 2008;21(1):109‐120. doi:10.1007/s10278-007-9017-8 17356803 PMC3043825

[28] Pfeiffer F , Weitkamp T , Bunk O , David C . Phase retrieval and differential phase‐contrast imaging with low‐brilliance X‐ray sources. Nat Phys. 2006;2(4):258‐261. doi:10.1038/nphys265

[29] Urban T , Noichl W , Engel KJ , Koehler T , Pfeiffer F . Correction for X‐ray scatter and detector crosstalk in dark‐field radiography. IEEE Trans Med Imaging. 2024;43(7):2646‐2656. doi:10.1109/TMI.2024.3374974 38451749

[30] Noichl W , De Marco F , Willer K , et al. Correction for mechanical inaccuracies in a scanning Talbot‐Lau interferometer. IEEE Trans Med Imaging. 2024;43(1):28‐38. doi:10.1109/TMI.2023.3288358 37342956

[31] Schick RC , Koehler T , Noichl W , et al. Correction of motion artifacts in dark‐field radiography of the human chest. IEEE Trans Med Imaging. 2022;41(4):895‐902. doi:10.1109/TMI.2021.3126492 34748485

[32] De Marco F , Willer K , Gromann LB , et al. Contrast‐to‐noise ratios and thickness‐normalized, ventilation‐dependent signal levels in dark‐field and conventional in vivo thorax radiographs of two pigs. PLoS One. 2019;14(6):e0217858. doi:10.1371/journal.pone.0217858 31158251 PMC6546243

[33] Sun W , Niessen WJ , Klein S . Hierarchical vs. simultaneous multiresolution strategies for nonrigid image registration. In: *Biomedical Image Registration. WBIR 2012*. Berlin, Heidelberg. Springer; 2012:60‐69. doi:10.1007/978‐3‐642‐31340‐0_7

[34] Qiu H , Qin C , Schuh A , Hammernik K , Rueckert D . Learning diffeomorphic and modality‐invariant registration using B‐splines. Proc Mach Learn Res. 2021;143:645‐664.

[35] Modat M , Daga Ridgway P , Cardoso MJ , et al. Parametric non‐rigid registration using a stationary velocity field. In: *2012 IEEE Workshop on Mathematical Methods in Biomedical Image Analysis*. Breckenridge, CO, USA. IEEE; 2012:145‐150. doi:10.1109/MMBIA.2012.6164745

[36] Avants BB , Epstein CL , Grossman M , Gee JC . Symmetric diffeomorphic image registration with cross‐correlation: evaluating automated labeling of elderly and neurodegenerative brain. Med Image Anal. 2008;12(1):26‐41. doi:10.1016/j.media.2007.06.004 17659998 PMC2276735

[37] Rueckert D , Schnabel JA . Medical image registration. In: *Biomedical Image Processing*, Berlin, Heidelberg. Springer; 2010:131‐154. doi:10.1007/978-3-642-15816-2_5

[38] Gassert FT , Urban T , Kufner A , et al. Dark‐field X‐ray imaging for the assessment of osteoporosis in human lumbar spine specimens. Front Physiol. 2023;14:1217007. doi:10.3389/fphys.2023.1217007 37534364 PMC10393038

[39] Rueckert D , Sonoda LI , Hayes C , Hill DLG , Leach MO , Hawkes DJ . Nonrigid registration using free‐form deformations: application to breast MR images. IEEE Trans Med Imaging. 1999;18(8):712‐721. doi:10.1109/42.796284 10534053

[40] Park JS , Oh SJ . A new concave hull algorithm and concaveness measure for n‐dimensional datasets. J Inf Sci Eng. 2012;28(3):587. doi:10.6688/JISE.2012.28.3.10

[41] De Vos BD , Berendsen FF , Viergever MA , Sokooti H , Staring M , Išgum I . A deep learning framework for unsupervised affine and deformable image registration. Med Image Anal. 2019;52:128‐143. doi:10.1016/j.media.2018.11.010 30579222

[42] Schuh A , Qiu H , HeartFlow Research. deepali: image, point set, and surface registration in PyTorch. 2024. doi:10.5281/zenodo.8170161

[43] Bech M , Bunk O , Donath T , Feidenhans R , David C , Pfeiffer F . Quantitative X‐ray dark‐field computed tomography. Phys Med Biol. 2010;55(18):5529. doi:10.1088/0031-9155/55/18/017 20808030

[44] Pierce RJ , Brown DJ , Holmes M , Cumming G , Denison DM . Estimation of lung volumes from chest radiographs using shape information. Thorax. 1979;34(6):726‐734. doi:10.1136/thx.34.6.726 542910 PMC471178

[45] De Winter JCF , Gosling SD , Potter J . Comparing the Pearson and Spearman correlation coefficients across distributions and sample sizes: A tutorial using simulations and empirical data. Psychol Methods. 2016;21(3):273‐290. 27213982 10.1037/met0000079

[46] Wielpütz MO , Heußel CP , Herth FJF , Kauczor HU . Radiological diagnosis in lung disease: factoring treatment options into the choice of diagnostic modality. Dtsch Arztebl Int. 2014;111(11):181‐187. doi:10.3238/arztebl.2014.0181 24698073 PMC3977441

[47] Tsalafoutas IA , Koukourakis GV . Patient dose considerations in computed tomography examinations. World J Radiol. 2010;2(7):262‐268. doi:10.4329/wjr.v2.i7.262 21160666 PMC2999328

[48] Mettler FA Jr , Huda W , Yoshizumi TT , Mahesh M . Effective doses in radiology and diagnostic nuclear medicine: a catalog. Radiology. 2008;248(1):254‐263. doi:10.1148/radiol.2481071451 18566177

[49] Schick RC , Bast H , Frank M , et al. Simulated low‐dose dark‐field radiography for detection of COVID‐19 pneumonia. PLoS One. 2024;19(12):e0316104. doi:10.1371/journal.pone.0316104 39729472 PMC11676568

[50] Petersson J , Rohdin M , Sánchez‐Crespo A , et al. Regional lung blood flow and ventilation in upright humans studied with quantitative SPECT. Respir Physiol Neurobiol. 2009;166(1):54‐60. doi:10.1016/j.resp.2009.01.008 19429519

[51] Dorosti T , Schultheiss Sheikh M , Schmette P , et al. Estimating total lung volume from pixel‐level thickness maps of chest radiographs using deep learning. Radiol Artif Intell. 2025; e240484. doi:10.1148/ryai.240484 40434310

