# Supplementary material for: Deformable image registration of dark‐field chest radiographs for functional lung assessment
Source: Med Phys. 2025 Aug 8;52(8):e18023. doi: 10.1002/mp.18023 (PMC12334873; doi:10.1002/mp.18023)
Supplement: Supplementary file 1 — Supporting Information [file MP-52-0-s001.pdf]

Supporting Information

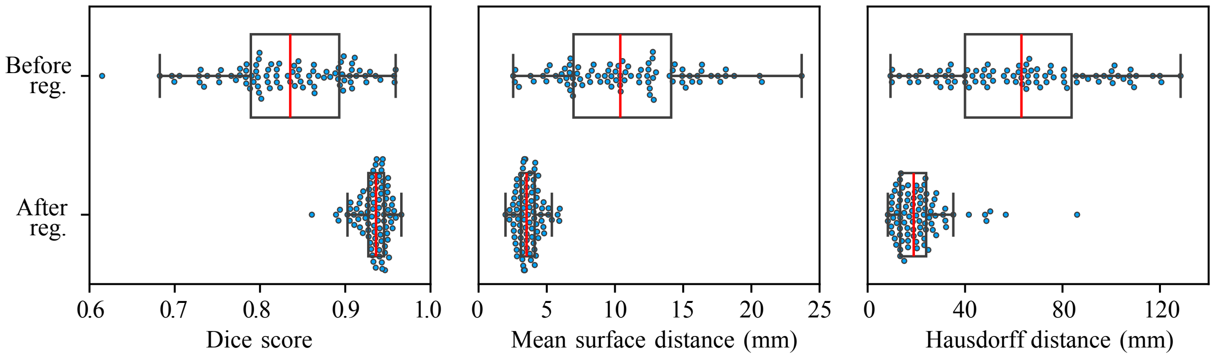

Figure S1: Registration evaluation metrics considering partial lung masks for the dark-field image registration framework within the dataset. The top row shows the scores before, and the bottom row the values after the registration procedure. Red lines indicate the median, black boxes the interquartile range (25th to 75th percentile), and blue dots the individual study participant scores.
